# Cockatiels sing human music in synchrony with a playback of the melody

**DOI:** 10.1371/journal.pone.0256613

**Published:** 2021-09-03

**Authors:** Yoshimasa Seki

**Affiliations:** Department of Psychology, Aichi University, Toyohashi, Japan; Texas Christian University, UNITED STATES

## Abstract

It is known among aviculturists that cockatiels imitate human music with their whistle-like vocal sounds. The present study examined whether cockatiels are also able to sing “in unison”, or, line up their vocalizations with a musical melody so that they occur at the same time. Three hand-raised cockatiels were exposed to a musical melody of human whistling produced by an experimenter. All the birds learned to sing the melody. Then, two out of these three birds spontaneously joined in singing during an ongoing melody, so that the singing by the bird and the whistling by the human were nearly perfectly synchronous. Further experiments revealed that the birds actively adjusted their vocal timing to playback of a recording of the same melody. This means cockatiels have a remarkable ability for flexible vocal control similar to what is seen in human singing. The proximate/ultimate factors for this behavior and implications for musicality in humans are discussed.

## Introduction

Humans have a large variety of complex songs and instrumental music, which makes human “musicality” special. Here, musicality is defined as the set of capabilities and proclivities that allows our species to generate and enjoy music in all of its diverse forms [1] and encompasses the underlying biological capacities that allow us to perceive and produce music [2]. In addition, singing along to an ongoing melody of familiar music is also widely seen in people. For example, at a birthday party when one person initiates the song “Happy Birthday”, it is easy for people to follow along [3], or to sing in unison (*i*.*e*., multiple individuals singing a single acoustic pattern in synchrony, not merely a temporal overlap of vocalizations produced by multiple individuals). However, singing in unison requires controlling outputs from the vocal organs to match both the timing and the spectral patterns of an ongoing sound stream; *i*.*e*., temporal and spectral synchronization [4]. That is not all. Musical melodies are not innately programmed into our vocal repertoires; thus, to join in an ongoing melody, it is necessary to encode and store the global structure of the melody in memory. Then, at the onset of singing, the singer must choose the appropriate starting note from memory instantaneously in order to coordinate vocal outputs along the sound stream. Therefore, complicated cognitive mechanisms should be involved in singing a musical melody in unison.

So, the question here is whether non-human animals are capable of showing a similar behavior. As described below, a few species sing songs imitating human music and even fewer animal species vocalize in unison. However, no study has reported these two capabilities exhibited together in non-human animals, to my knowledge. The present study demonstrates that cockatiels are able to imitate the melody of (*a*) human music and are able to sing it (*b*) in unison (*c*) spontaneously (*d*) with playback of recorded sounds performed by a human (*i*.*e*., not conspecifics) (*e*) in a context apart from reproduction. To underscore the significance of the results, some relevant points are introduced in the following paragraphs.

### Imitation of human music by non-human animals

Some non-human animals, such as songbirds, can add novel acoustic patterns to their own vocal repertoires by listening to sounds [5, 6]; they are considered vocal learners. However, in most vocal learning animals, the innate constraints for incorporating novel sounds is much stricter than in humans. For example, juvenile finches learn songs from their father very well; however, they copy songs imperfectly when the tutor is a foster father of a different species [7, 8]. Therefore, it makes sense that most animals do not imitate melodies of human music.

Some vocal learning animals imitate human speech and/or artificial sounds. The African grey parrot, Alex [9], and the harbor seal, Hoover [10], may be the most famous examples. More recently, a study reported that an elephant produced human speech sounds [11]. Some vocal learning animals also imitate various artificial sounds. Lyrebirds imitate the noises of a chainsaw [12], for example. However, human music has unique qualities apart from speech and other artificial sounds; music has harmonic syntax, rhythmic syntax, and is characterized by meter, grouping and hierarchy. Some of these qualities are shared in common with language, but not all of them [13]. Thus, the cognitive processes involved in musicality should differ from those used in the perception and production of other types of sound sequences, including human speech. Moreover, it is plausible that the acoustic structure of human music is quite different from the acoustic structure of natural animal vocalizations. Thus, musical melodies may be suitable imitation models for evaluating the flexibility of cognitive processing involved in vocal learning in animals. Therefore, investigating whether animals can imitate human music may be an interesting research theme, and may provide more insight for further understanding of their cognitive capabilities, beyond whether they can imitate human speech and/or artificial noises.

Anecdotally, many videos have been uploaded to online databases (such as YouTube) by aviculturists, in which cockatiels imitate melodies of human music with their whistle-like vocal sequences. Examples include the “Mickey Mouse Club March” and the theme song of “My Neighbor Totoro”. However, there are only a few academic publications describing imitation of human music by animals. One study showed that bullfinches imitated human music and sang the melody alternately with whistling produced by an experimenter [14]. Further, it was documented that a European starling named Kuro sang some melodies of human music [15]. Another study reported that gray seals learned to modify their vocalizations to match the frequency patterns of musical melodies that were composed of human vowel-like sounds under an operant conditioning paradigm [16] (*note*: in these studies, animals did not sing in unison). In sum, further academic documentation of music production by animals would be valuable for comparative approaches in relevant research fields, such as neuroscience, cognitive studies, and psychology.

### Singing in unison by non-human animals

Many animals, including non-vocal learners and even invertebrates, sing songs together, forming duets or choruses [17]. As an example, howling by wolves results in acoustic patterns that form a heterophony [3]. As another example, two gibbons generate great calls together with the vocal timing of one individual likely depending on that of the other [18–20]. In addition, many studies documented songbirds singing together [21]. Songbirds are vocal learners; thus, singing together in songbirds is much more relevant to the present topic than similar behaviors in non-vocal learners. However, studies demonstrating singing *in unison* or, *monophony* (as opposed to simply singing at the same time) by non-human animals are quite rare, including those in songbirds. Likely, this is one reason why one author stated that “human beings are the only species to have evolved the ability to sing in unison in both the dimensions of rhythmic co-ordination and precise pitch attunement” [22]. As a matter of fact, it is known that a few avian species do sing in unison. Male and female mates of the forest weaver sing in unison, which were likely derived from a territorial display [23, 24]. White-browed sparrow weavers also sing in unison [25]. Lastly, male and female plain-tailed wrens sing duets antiphonally, but occasionally males and females sing songs in unison with near perfect synchrony [26–28]. In mammals, recently, a study reported that two male dolphins occasionally produced isochronous pop sound sequences in synchrony [29], which is likely the only academic report of non-human mammals vocalizing in unison thus far. However, this behavior is much simpler than the singing in unison performed by humans.

In sum, the examples of singing in unison by non-human animals reported in these studies were (*i*) only between conspecifics, (*ii*) occurred in specific contexts involving reproduction (including territorial defense), and (*iii*) used only vocal variations observed in the wild (the vocal system is optimized to use those sounds, making the behavior relatively easy; on the contrary, learning and producing a melody of human music may require much larger costs for the vocal system as described above). Therefore, the present study would provide further knowledge to understand the similarities and differences seen between humans and non-human animals with regard to musicality.

### Introduction for the experiment

A research project was launched to investigate musicality in non-human animals. Cockatiels were chosen as the subjects because it is well known that they sing melodies of human music as described above. In the project, three hand-raised cockatiels learned to sing a marching sound: whistling of a melody similar to the “Mickey Mouse Club March” produced by a human (the experimenter). The melody was composed of the two parts; the first half (consisting of 11 notes) and the second half (consisting of 11 notes) separated by a long pause (640 ms, see Materials and Methods). Then, two of the birds (bird C and bird PY) spontaneously sang in unison with the whistling (see S1 Movie). Therefore, the following two experiments (with some predictions) were carried out to examine whether the birds would actively synchronize their vocalizations with a playback of a recording of the melody.

#### Experiment 1

A playback sequence is presented while a bird is singing to observe how the bird would modulate his vocal timing.

#### Prediction I

If a bird actively synchronizes his vocal timing to the playback sound, he will do so by lengthening the duration of the long pause between the first half and the second half. This long pause between the halves of the melody provides a good opportunity for the bird to adjust his vocal timing with the playback sound sequence. If the delay between the bird’s singing and the start of the playback increases, the bird will compensate by also increasing the duration of the pause between the two melody halves.

(**Alternative**) If the duration of the long pause remains the same regardless of the playback delay, the time difference between the playback and singing will be maintained until the end of the melody. This suggests that the bird did not actively synchronize to the melody.

#### Experiment 2

A playback sequence is presented when a bird is not singing to observe whether the bird begins to sing following the playback, and how he modulates his vocal timing to synchronize with the playback of the melody.

#### Prediction II

If the bird begins to sing following the playback, he will start singing in the middle of the melody at any time point along the ongoing sound sequence, synchronizing his vocal timing with the playback. As a result, the song will be an irregular sequence because it lacks some of the initial notes.

(**Alternative**) If the bird always starts singing from the beginning of the song, even when the playback has already advanced past this point in the melody, it suggests the bird ignored the playback sound and did not synchronize his song to the playback melody.

#### Prediction III

If the bird begins to sing following the playback and begins the song from the initial note (instead of the middle of the melody as in Prediction II), the song is slightly delayed from the playback melody. Therefore, if the bird actively synchronizes to the melody, he may skip some of the latter notes in the first half in order to adjust his vocal timing to the playback at the onset of the second half of the melody. This will also result in an irregular song sequence. While there are other ways in which the bird could adjust his vocal timing (*e*.*g*., singing the entire first half and shortening the duration of the pause between melody halves; however, this requires reducing a period of rest and rushing to vocalize the second half of the melody. So, this strategy is not likely), this is a likely scenario.

(**Alternative**) If the song sequence is unchanged, and all notes are present, it suggests the bird ignored the playback sound and did not synchronize his song to the playback melody.

In the experiments, both playback sounds and vocal sounds of the birds were simultaneously recorded to analyze whether the birds sang as predicted.

## Results

### Experiment 1: *A playback sequence was presented while a bird was singing*

**Fig 1:**
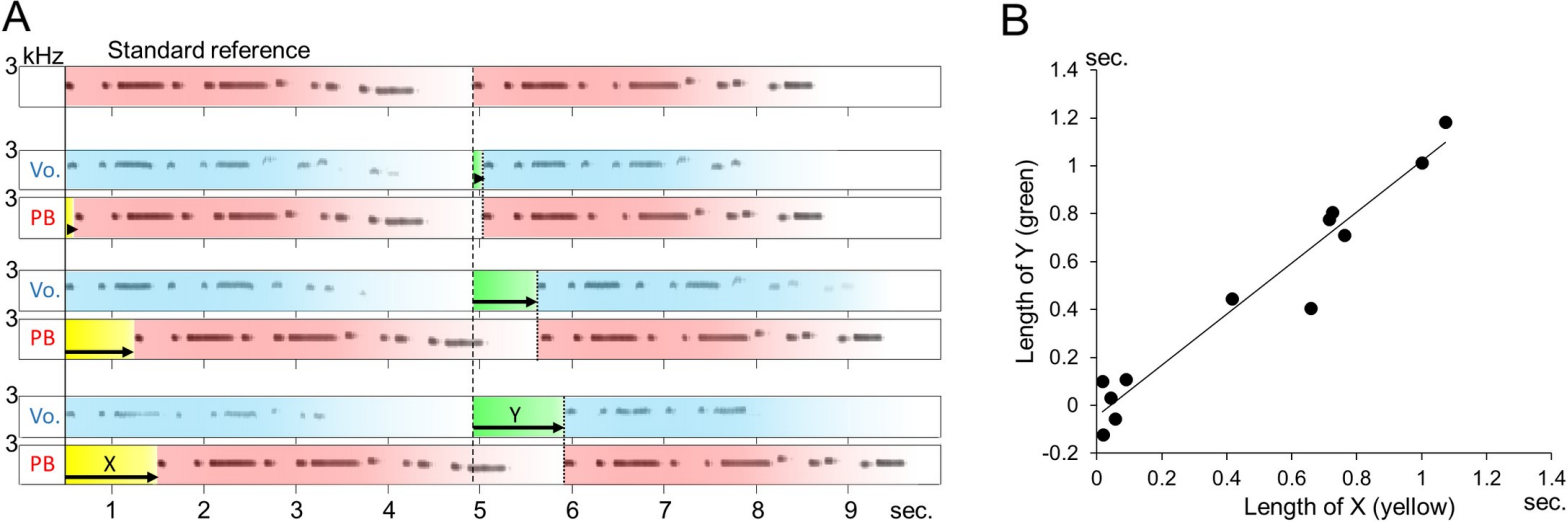
Effects of playback sounds on ongoing singing. These recordings were obtained from bird C (the same recordings are shown as C#3, C#4 and C#6 in *S1 File*). Model sounds (PB: playback, pink shading) were played back after the bird began singing (Vo.: blue shading). Yellow shading indicates the latency of the playback sound (X). Green shading indicates the duration between the onset of the second half of the standard reference (see main text) and the actual onset of the second half of singing (Y). Note that the onset of the second half of the playback sound and the singing started at nearly the same time (A). Correlation between the length of X and the length of Y is shown in Fig 1A. Each dot denotes values of X and Y obtained from a recording that includes bird singing and playback of the melody. When the length of X is longer, the length of Y is longer (B).

Using the updated recording system (see Materials and Methods), 7 recordings were obtained from bird C (C#1 − C#7 in Supplementary Information; hereafter, *S1 File*) and 5 recordings were obtained from bird PY (PY#1 − PY#5 in *S1 File*), in which the birds kept singing after the playback sounds were presented. The birds changed the pause duration of their own song (consistent with ***Prediction I***). The actual onset of the second half of singing was delayed from the putative onset (the original playback sound was used as a standard reference to measure the delay; see Fig 1A upper and *S1 File*). The delay in singing (Y; indicated by green shading in Fig 1A) depended on the latency of the playback (X; indicated by yellow shading in Fig 1A). The duration of Y was longer when X was longer (Fig 1B). There was a significant correlation between X and Y (*r* = 0.972 [95% CI = 0.899 − 0.992], *t* = 12.975, df = 10, *p* < 0.001; individual results are also shown in *S1 File*). These results indicate that the birds actively adjusted their vocal timing to the playback sound.

### Experiment 2: *A bird began to sing following the presentation of a playback sequence*

**Fig 2:**
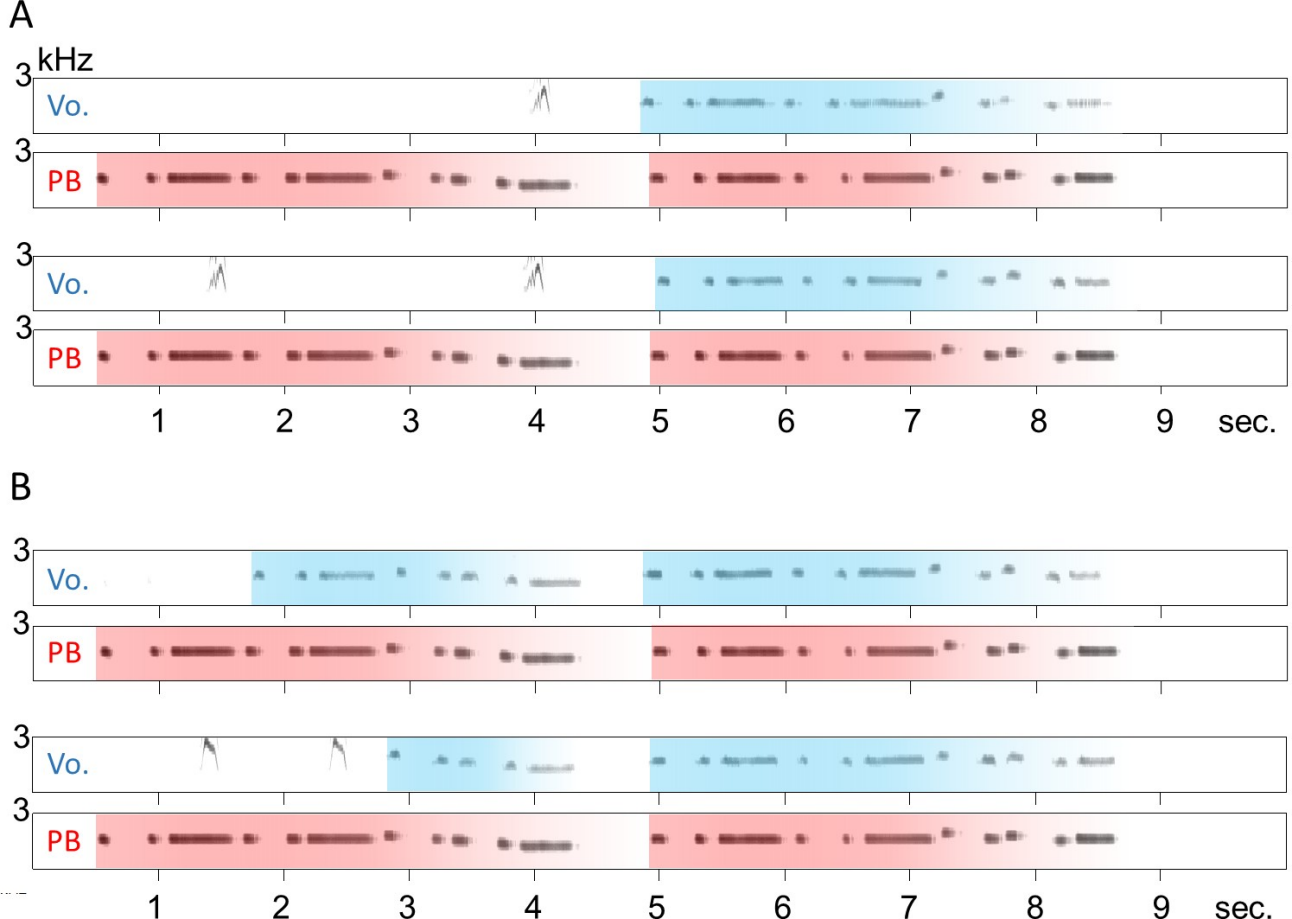
Examples of singing in response to playback sounds. These recordings were obtained from bird PY. Starting from the second half (A; the same recordings are shown as PY#6 and PY#10 in *S1 File*). Starting from in the middle of the first half (B; the same recordings are shown as PY#12 and PY#13 in *S1 File*).

Bird C did not begin to sing following the presentation of a playback sequence. However, 15 recordings were obtained from bird PY, in which the bird sang songs following the presentation of playback sounds (PY#6 − PY#20 in *S1 File*). In 1 out of the 15 recordings, the bird sang the normal sequence of the melody with some irregular note intervals, as if he was trying to ignore the playback sound; then, he stopped singing in the middle of the second half (PY#18 in *S1 File*). In 2/15 recordings, the bird sang a limited part of the first half and then stopped singing suddenly (PY#19 and PY#20 in *S1 File*). However, in 9/15 recordings, the bird started singing and synchronized his vocal timing with the playback, either from the beginning of the second half by skipping the entire first half (5/15 recordings; Fig 2A; PY#6 − PY#10 in *S1 File*), or from the middle of the first half by skipping several (between 1 and 6) initial notes (4/15 recordings; Fig 2B; PY#11 − PY#14 in *S1 File*). Both of these adjustments are consistent with ***Prediction II***. The bird occasionally sang songs spontaneously during the experimental period, so 46 song recordings were obtained without the playback sound. In these recordings, the bird always began singing from the beginning of the first half and never started in the middle of the melody (0/46 recordings), indicating that any abnormal singing observed was not caused by chance, but was in response to the playback sound (*p* < 0.001, Fisher’s exact test).

**Fig 3:**
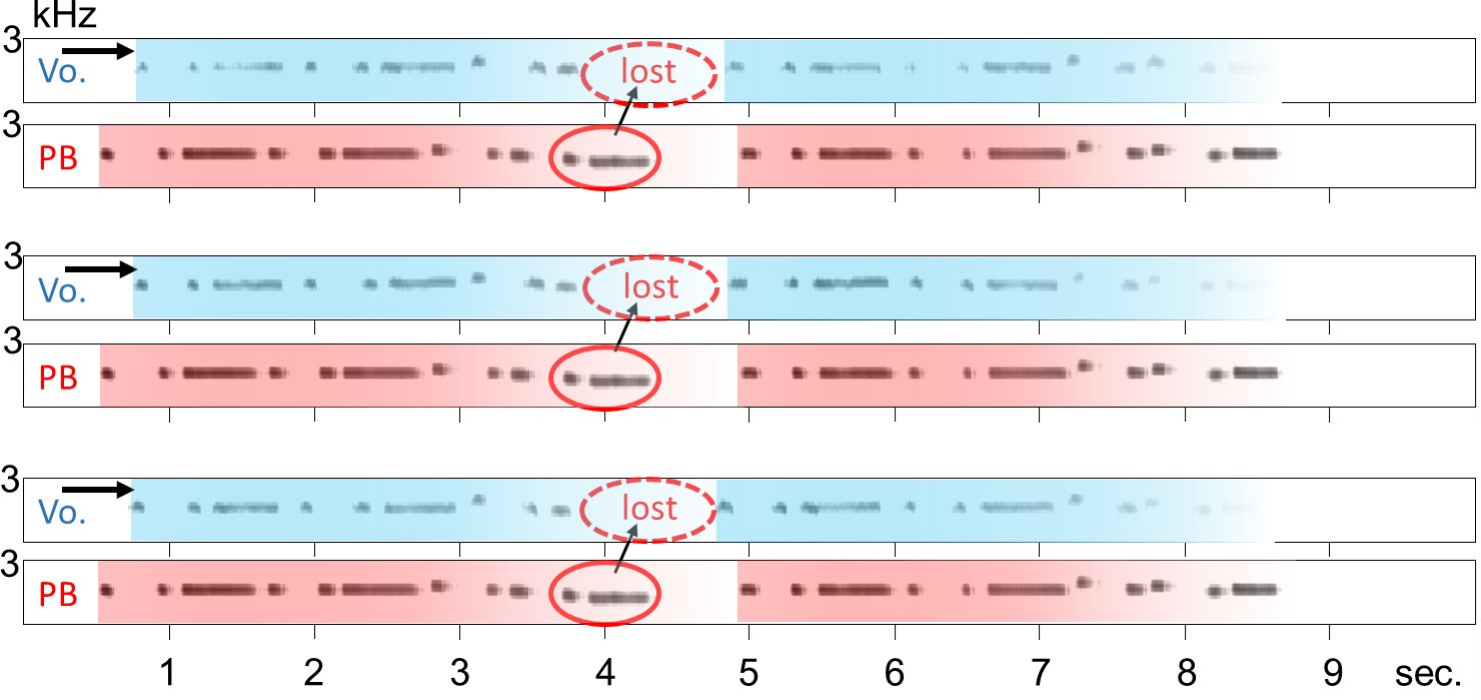
Examples of irregular vocal sequences with playback of the melody. These recordings were obtained from bird PY (the same recordings are shown as PY#15, PY#16 and PY#17 in *S1 File*). The vocalizations began following the playback (indicated by black arrows) and the last two notes of the first half were dropped (indicated by red circles).

In 5/15 recordings, bird PY started singing from the beginning of the sequence with a slight delay following a playback sound (PY#15 − PY#19 in *S1 File*). Each time, the latency was roughly the same value (mean = 243.9 ms, SD = 20.99 ms). Therefore, it is possible that these 5 songs were triggered by the first note of the playback sound. While the bird stopped singing in the middle of the normal song in 2 out of the 5 recordings (PY#18 and PY#19 in *S1 File*), he sang the song to the end in 3/5 recordings (Fig 3; PY#15, PY#16 and PY#17 in *S1 File*). However, in these 3 recordings, the last two notes of the first half were dropped and the songs were resumed at the onset of the second half, which did not happen when the bird was singing without the playback (0/46). Thus, it is likely that this behavior did not simply occur by chance (*p* = 0.029, Fisher’s exact test), suggesting the bird actively skipped those two notes to synchronize his vocal timing with the playback at the beginning of the second half. This adjustment is consistent with ***Prediction III***.

In Experiment 2, there were several examples in which Bird PY began to sing following the playback and it is possible that the bird entrained to the ongoing melody. If this is the case, then for the 12 recordings which were consistent with our predictions (9 songs for Prediction II and 3 songs for Prediction III) we might see a negative mean asynchrony (NMA). It is well known that when humans create a series of motor outputs synchronizing with a rhythmic stimulus, their motor outputs tend to slightly precede each stimulus presentation, which is termed NMA [30]. Also, NMA is considered one indication that animals have entrained to a rhythmic sequence [31]. To examine this possibility, the timing of the vocalization at the onset of the second half was compared to the timing of the playback at the onset of the second half. The results showed that singing started slightly but significantly faster than the playback (mean -135 ms, median -94 ms; V = 8, *p* = 0.012, Wilcoxon signed rank test [the null hypothesis; mu = 0]), which can be interpreted as an NMA. This result also supports the interpretation that the bird actively synchronized his vocal timing with the playback.

## Discussion

The results indicate that the cockatiels actively synchronized their vocal timing with the playback of the melody of human music. As described in the Introduction, imitation of human music was previously reported in only a few non-human animal species. Likewise, academic reports for singing in unison by non-human animals are rare. Therefore, this is the first example, to my knowledge, of vocal performance by non-human animals combining these two abilities (singing a melody of human music and synchronizing to a model sound). In addition, the cockatiels sang spontaneously with a non-conspecific animal (*i*.*e*., a human), without food rewards, and in a non-reproductive context. Given these conditions, it is likely that this experimental demonstration can provide some novel insights for this field of research. In addition, the results suggest the cockatiels’ ability for processing a series of complex neural commands; that is, they are able to memorize the whole melody, determine where the note is located within the ongoing melody, and then control their vocal outputs with precise timing. However, it is important to state that the present study did not examine the capability of the birds to modulate spectral patterns in response to frequency-altered playback of the model sound (*i*.*e*., spectral synchronization), which would be an interesting challenge for future study.

### Why do cockatiels sing in unison?

To obtain an answer to this question, we can turn to previous studies investigating the vocal behavior of wild parrots, but there are only a few studies. Duets between conspecifics (sung in antiphony, not in unison) have been reported in wild Grey-headed parrots [32] and Yellow-naped amazons [33]. The duets of Yellow-naped amazons are produced by a male and female pair, which are sung for joint territory defense [34]. However, the behavioral contexts in which these wild parrots sing duets differ from the contexts for singing in unison by the cockatiels in this study.

As some authors described, studying the vocal learning of parrots in the wild is difficult due to several reasons: they stay high up in the canopy, move in and out of the foliage, and do not generally stay in one place for long periods of time [35, 36]. Thus, there are also currently no academic reports of vocal learning in wild cockatiels, to my knowledge. Moreover, there are few laboratory studies examining the vocal behavior of cockatiels, most of which describe only their short calls, and not songs [37–41]. As one exception, an author described that captive cockatiels often produced complex, long-lasting vocalizations which might appear to qualify as song [42]; however, the author did not describe the acoustic details or the function of these vocalizations. It is known among aviculturists that captive cockatiels often imitate human speech, music and other sounds; however, it is unclear whether cockatiels sing songs in the wild or not (and consequently, even if they do, the function is unknown). To the contrary, budgerigars very often sing warble songs. It is assumed that the songs are used for bonding among social companions, individual / group recognition, and as a badge of group membership [43]. Therefore, the speculation that cockatiels sing in unison for social bonding may be valid. Anecdotally, the cockatiels in this study often sang the musical sounds or imitated human speech when caregivers were leaving the aviary. Therefore, they may have done so to attract attention from humans, though there is no quantitative data to support this observation. This is consistent with the music and social bonding hypothesis [2]. This idea is also supported by the fact that no food reinforcements were necessary for the birds to sing in unison. We may think about another related question. The bullfinches [14] and European starling Kuro [15] described in the Introduction were hand-reared, as were the cockatiels in the present study. Therefore, these birds might recognize their human caregivers as their own conspecifics, and might recognize human music as vocalizations produced by conspecifics. As a result, they might acquire the melodies in the same way as many songbirds acquire songs from conspecifics. On the contrary, it is also possible that the birds learned the melody even though they recognized their human caregivers as heterospecfic animals and considered the melodies to be heterospecific vocalizations. However, there is currently a lack of quantitative data enabling further discussion of these possibilities. We must wait until the points described above have been tested experimentally.

### How do cockatiels sing in unison?

Auditory-vocal mirroring in the neural circuit involved in vocal production and vocal learning in songbirds may partially explain the substrates for this behavior. Some neurons in the nucleus HVC (proper name, not an abbreviation) are activated not only when a bird produces a particular song note but also when the bird listens to the same song note [44]. Parrots have a neural circuit for vocal production and vocal learning similar to songbirds that contains the nucleus NLC (central nucleus of the lateral nidopallium) which corresponds to the songbird HVC [45]. Therefore, it is valid to speculate that some neurons involved in the production of the melody were also activated when birds listened to the playback of the melody as if the cockatiel was singing. The neural activity elicited from listening might lead to singing of the melody. Thus, the present findings may be a behavioral demonstration suggesting parrots have a neural system for auditory-vocal mirroring similar to songbirds. Another recent study recorded neural activity of HVC neurons in songbird pairs during duet singing. The authors reported that the degree of interindividual synchronization of neural activity was positively correlated with the degree of interindividual synchronization of vocal activity during alternating parts of duet bouts [46]. Therefore, the activity of NLC in cockatiels might be involved in the timing control of singing in unison.

Further, parrots have additional structures in their vocal learning nervous system which may be involved in this behavior. The parrot vocal nuclei have core regions and shell regions. Connections between the cores and shells are sparse within and among each vocal nucleus. Cores tend to project to cores, and shells tend to project to shells. This results in two parallel systems: a core system and a shell system [47]. The neural connectivity of the core vocal nuclei is similar to that of the song nuclei of songbirds, whereas the shell system is unique to parrots. The unique system may allow for more complex vocal communication abilities and greater auditory–motor entrainment than in other birds [48].

In addition, parrots may have excellent neural and psychological substrates to synchronize their motor outputs to external rhythmic stimuli. The cockatoo, Snowball, spontaneously showed entrainment through dance to various beats of human music [49], which was considered the first demonstration of this type of behavior by non-human animals. Other studies trained budgerigars to synchronize to metronomic sounds using operant conditioning techniques [31, 50]. While the impact of these budgerigar studies are less than that of the Snowball study, the results support the idea that some parrot species have an excellent capability for entrainment. Rhythmic entrainment should be one of the important factors required for singing in unison; thus, the neural and psychological substrates for rhythmic synchronization of body movements may have a linkage to that for rhythmic synchronization of vocalizations, at least in parrots.

In addition, some recent studies suggest that parrots may have some further capabilities involved in musicality. Snowball originally created diverse motor patterns for dancing that were performed with musical melodies [51]. A study reported that an African grey parrot was trained to produce a vocal sequence following a sound sequence produced from a piano. Each sequence produced by the bird followed musical rules similar to the previous piano sequence in terms of the frequency ratios between the notes [52]. Another study showed that wild palm cockatoos can make drumming sequences with regular intervals using several tools [53]. Moreover, a recent study reported that cockatiels manipulated several objects producing sounds [54]. The substrates for singing human music in unison by cockatiels may also be relevant to these behaviors.

#### Implications of singing in unison by non-human animals for understanding musicality

Modern human society is full of music. We often listen to music and play music in various situations apart from sexual and reproductive contexts. Nevertheless, it does not mean that people find just any complex sound sequence pleasing to the ear. Humans have a preference for the structure of musical sounds, such as harmonic intervals (or, consonance). The harmonic intervals used in music are mostly consistent across different cultures [55]. In consonant melodies (*e*.*g*., consisting of minor and major thirds [56]), pitch classes often reflect small-integer frequency relationships (in the above example, minor and major thirds are 5:6 and 4:5 frequency ratios, respectively) and unison makes the simplest frequency ratio (*i*.*e*. 1:1) [2]. Therefore, while cockatiels are singing in unison, they are creating a consonant melody with their vocalizations. Here, we can consider the question of why some non-human animal species prefer consonance while other species do not [56–58]. The vocal similarity hypothesis [55] suggests that, in humans, the preference for consonance in music may originate from its similarity to the acoustical structure of their voice. Further, non-human animals are likely to use relative pitch to identify harmonic information that is similar to their own vocalizations [59]. From this perspective, it is valuable to investigate whether there is a preference for consonant sounds in the cockatiels, because they spontaneously created a consonant melody while singing in unison. Thus, if they prefer consonant sounds, it may support the vocal similarity hypothesis. Additionally, aligning acoustic spectra can promote bonding in humans (*e*.*g*., people would get along with each other through singing in a chorus group or playing instruments in a band/orchestra) [2]. So, cockatiels singing in unison with human whistling may be involved in social bonding (*i*.*e*., creating social relationships between cockatiels and humans). Lastly, in singing in unison, cockatiels likely prefer to imitate human music performed with whistle sounds rather than human speech, even though they can imitate various human speech sounds. Thus far, I have never observed cockatiels “talking” in unison with human speech sounds. This might be consistent with the argument that the processing of pitch information differs significantly for speech and music in humans [60]. As another possibility, when cockatiels vocalize in unison, they may prefer the higher frequencies used for imitating whistle sounds to the lower frequencies used for imitating human speech. In any case, altogether, the present findings append further perspectives on the recent progress in comparative approaches for investigating human musicality [61–66].

## Materials and methods

All experimental procedures and housing conditions were approved by the Animal Experiments Committee of Aichi University (approval number 15–01). All experiments were performed in accordance with the Fundamental Guidelines for Proper Conduct of Animal Experiments and Related Activities in Academic Research Institutions under the jurisdiction of the Ministry of Education, Culture, Sports, Science and Technology of Japan (MEXT).

### Subjects

Male cockatiels were obtained from a local breeder at the age of approximately 25 days post-hatch (dph) and were kept in independent cages (370 mm (W)× 415 mm (D) × 440 mm (H)) placed in an aviary (25°C, 12:12h photoperiod) at Aichi University. The birds were hand-raised by human caregivers to facilitate the ability to mimic sounds produced by humans and frequently heard human voices; however, the birds were isolated from human music except the model melody as much as possible (they might be occasionally exposed to some sounds, such as chimes and sirens, when they were outside their rearing room).

### Apparatus

Stimulus sounds (human whistles) and birds’ vocalizations were captured with a cardioid microphone (PRO35, Audio-technica, Japan) and recorded with a PCM recorder (DR-40, Teac, Japan). Stimulus sounds were presented through a loudspeaker (either AT-SP151 or AT-SP120, Audio-technica) during the playback experiment.

### Stimulus

**Fig 4:**
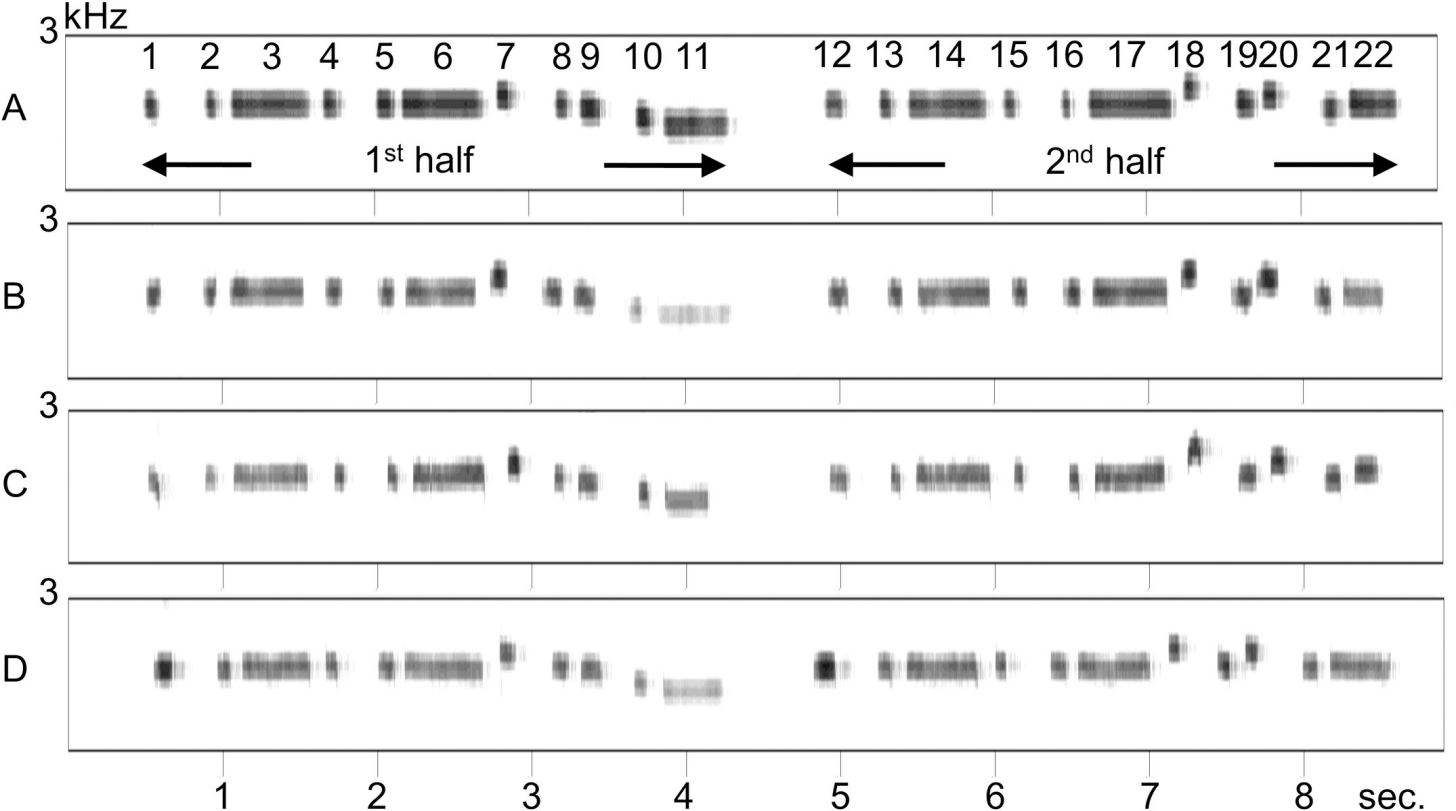
Example spectrograms showing imitation by the birds. The model sound (A) and vocalizations by bird PY (B), by bird C (C) and by bird PK (D). As shown in S3 Movie, the birds learned to imitate the melody step-by-step. These recordings were obtained soon after they began to sing the full melody. PK imitated the music, but did not sing in unison as frequently as the other two birds, so this bird did not participate in further experiments of this study.

A typical series of whistle sounds (which was similar to a portion of the “Mickey Mouse Club March”; 8.1s duration, 107 bpm [beats per minute], frequency range: 1100−1950 Hz) produced by the experimenter was recorded as a Windows PCM file (.wav format; 44.1 kHz sampling rate) and band-pass filtered (500–4000 Hz) using software (SASLab Pro., Avisoft Bioacoustics, Germany). The sound sequence was used as a template (or, a model) for the imitation by the birds. The melody was composed of 22 notes and was divided into two halves (11 notes each), which were separated by a long pause (640 ms; Fig 4A). Live performance of the whistle sounds was often presented to the birds (see below) to facilitate imitation. To human ears, the live sounds are nearly identical to the sounds in the recording. More importantly, the two sources of the melody seem to be identical to the birds, because they sang songs similarly in response to both the live performance (see S1 Movie) and the playback sounds (described below).

### Vocal learning of the melody

From early in life, three birds were exposed to the whistle sound sequence up to 30 times per day, either produced live by the experimenter or from a recording of the melody. The birds spent a large portion of each day with humans and were released from their cage for about 1 hour each day to play with humans. This was done to enhance their social relationship with humans, which could facilitate mimicry of the melody by the birds. Therefore, it was not feasible to keep birds inside an experimental box to record their vocal development as has been done in previous songbird studies [67]. Thus, I do not have a complete history for the vocal development of the birds and vocalizations were only recorded when birds produced sounds of interest (*e*.*g*. S2 and S3 Movies).

**Fig 5:**
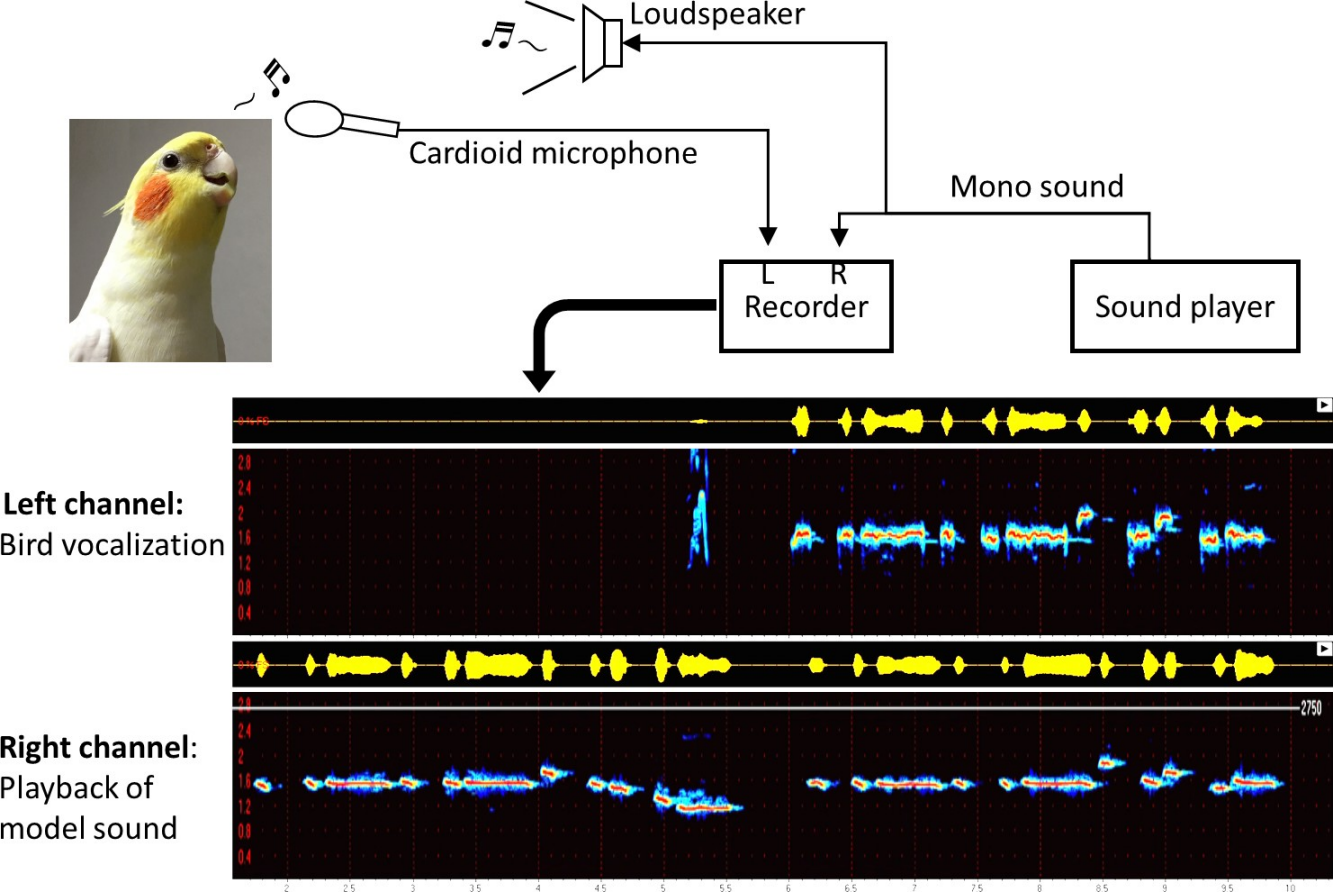
A schematic diagram of the updated recording system. All recordings of singing by the birds used for the analyses in Results section were obtained using this system. Therefore, the stimulus sounds presented to the birds were played back from the PC, not live performance by the experimenter.

Vocal imitation was established without any food rewards, though the human caregivers often verbally rewarded the birds for their vocal behavior (see S1 Movie). The birds were not trained with any specific methods, such as the model-rival method [9]. However, eventually, the birds were able to imitate the sounds (Fig 4B–4D, S4 Movie). Throughout this process, both live performance of whistles by the human and a recording of the whistle sounds played back from a PC (Fig 5, see below) were used in parallel as the model sounds. A number of songs were recorded in both the live and playback conditions; however, for the analyses in the Results section, only songs recorded with the playback sounds were used. This method ensures that the stimulus sounds were always consistent. Therefore, the active adjustment of vocal timing to a playback sound by the birds was quantitatively examined.

### Initial recordings via a single channel

A number of solo songs from the birds and vocal responses to the playback melody were recorded via the monaural channel of the recorder. In the latter phase of development (after the birds were nearly one year old), the recording began to include a considerable number of songs sung in unison with the melody. However, eventually it was difficult to sufficiently separate the vocal signals from the playback sounds in the single channel recordings for analysis. Nevertheless, human listeners could clearly identify the birds singing in unison (S5 Movie, for example). Therefore, a new recording system was built, which allowed for more rigorous analysis.

### Recordings with the updated system

Vocal sounds from the birds were recorded by the left channel of the PCM recorder via the microphone, which was directed toward the subject. The stimulus sound signals were sent out from Windows Media Player on a PC. The output was divided into two streams via audio connectors. One of those streams was amplified and presented to each bird as the sound stimulus through the loudspeaker (about 60 dB at the bird’s position) located at the insensitive direction of the microphone; the other was connected directly to the right channel of the PCM recorder. This setup enabled recording of both the birds’ vocalizations and the playback stimulus simultaneously and separately (Fig 5). All data shown in the Results section was recorded with this updated system. There were no other birds in the recording room, which was kept in silence (the background noise level was about 35 dB) during the experiments. Data used for analysis was recorded from each bird at the approximate ages of 330 dph (bird C) and 450 dph (bird PY). Recordings took place 1−2 hours a day for 6 days in total for each bird.

### Analyses

Birds’ vocalizations were edited and analyzed with SASLab. Low frequency sounds were cut off with a high-pass filter (0.5 kHz). The sound amplitude was normalized to 75% of the dynamic range at the peak amplitude of the sound sequence. The sound spectrograms (FFT length: 512, Temporal resolution: 87.5% overlap) were created and the onset of each note was obtained with a function of the software (Automatic parameter measurement).

### Statistics

Statistical analyses were performed using R3.4.1. Functions “cor.test” with Pearson’s correlation coefficient, “fisher.test” and “wilcox.exact” were used. All tests were performed two-sided.

## Supporting information

S1 Movie Singing in synchrony with human whistling by the birds. Example showing that birds C and PY spontaneously joined the music in the middle and synchronized to live whistle sounds produced by the experimenter (This movie is presented as an example and the songs were not used for the analyses in the Results).
(MP4)

S2 Movie Other vocal imitation by the birds. Example sounds and the spectrograms showing that birds spontaneously imitated not only the melody, but also several human words. For example, the birds often vocalized their own name.
(MP4)

S3 Movie Vocal development of the birds. By < 100 dph, the birds had already started producing vocal sequences which had similar characteristics to the model, at least to human listeners. The vocalizations gradually became more similar to the model sound, and by < 220 dph, the vocalizations could be clearly recognized by human observers as imitations of the model sounds.
(MP4)

S4 Movie Imitation of the melody by the birds. Example sounds and the respective spectrograms demonstrating imitation of the melody by the birds (the sound sources used to make Fig 4 and S4 Movie are identical).
(MP4)

S5 Movie An example of single channel recording. Another example of a sound sequence and the corresponding spectrogram showing singing in unison by bird PY (and live whistling by the experimenter). This was recorded from a single channel. Singing starts from the second half of the melody. Due to overlap of the two signals, it is difficult to separate vocal signals produced by the bird from the whistle sounds produced by the experimenter. Although a number of these types of recordings (both with live performance and with playbacks) were obtained, these data were not used for the analyses in the Results section due to the difficulty in separating the two signals.
(MP4)

S1 File This file includes all sound spectrograms used in the analyses. (PDF)
