# Supplementary material for: Cockatiels sing human music in synchrony with a playback of the melody
Source: PLoS One. 2021 Sep 3;16(9):e0256613. doi: 10.1371/journal.pone.0256613 (PMC8415583; doi:10.1371/journal.pone.0256613)
Supplement: S1 File — (PDF) [file pone.0256613.s006.pdf]

Supporting Information

for "Cockatiels sing human music in synchrony with a playback of the melody"

To measure the duration of Y, generally the length was measured using note #12 since this was the onset note for the second half of singing (see main text and Figure 1A). However, for 2 out of 6 songs by Bird C (C #1, C#2), the length was measured using note #13 (normally the second note of the second half of singing) because the 12th note was dropped from these 2 songs and Bird C started singing on note #13.

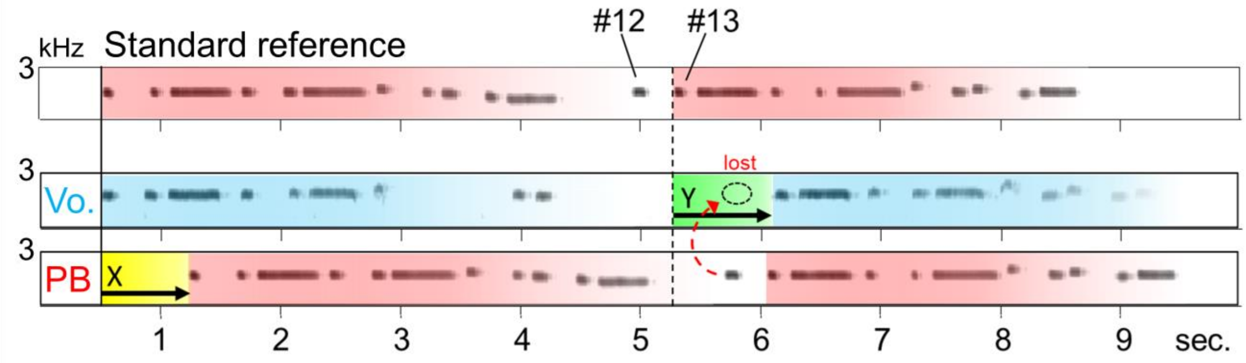

Sound spectrograms of all the data shown in the Results section of the main text (7 recordings from Bird C and 5 recordings from Bird PY [which are consistent with Prediction I]; 5 recordings from Bird PY [which are consistent with Prediction II (a)]; 4 recordings from Bird PY [which are consistent with Prediction II (b)]; 3 recordings from Bird PY [which are consistent with Prediction III]; and others) are displayed below:

(x-axes: time, y-axes: sound frequency [0−3kHz], Vo: vocalizations of the birds, PB: playback sounds)

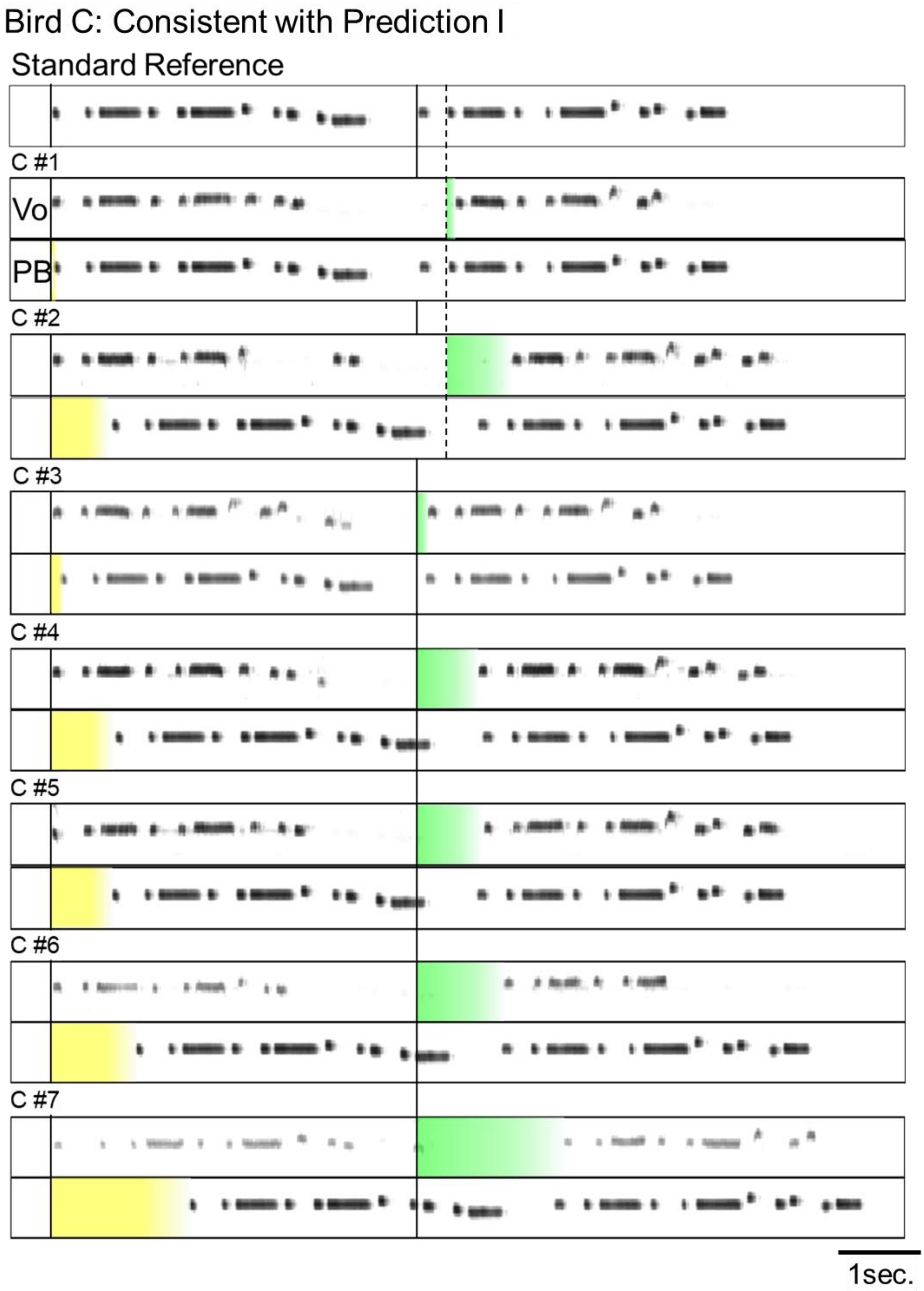

Recordings from bird C. Yellow shading indicates the latency of the playback sound (X). Green shading indicates the duration between the onset of the second half of the standard reference and the actual onset of the second half of singing (Y: see main text). Note, the bird sang the melody in synchrony with the playback when he was singing the second half.

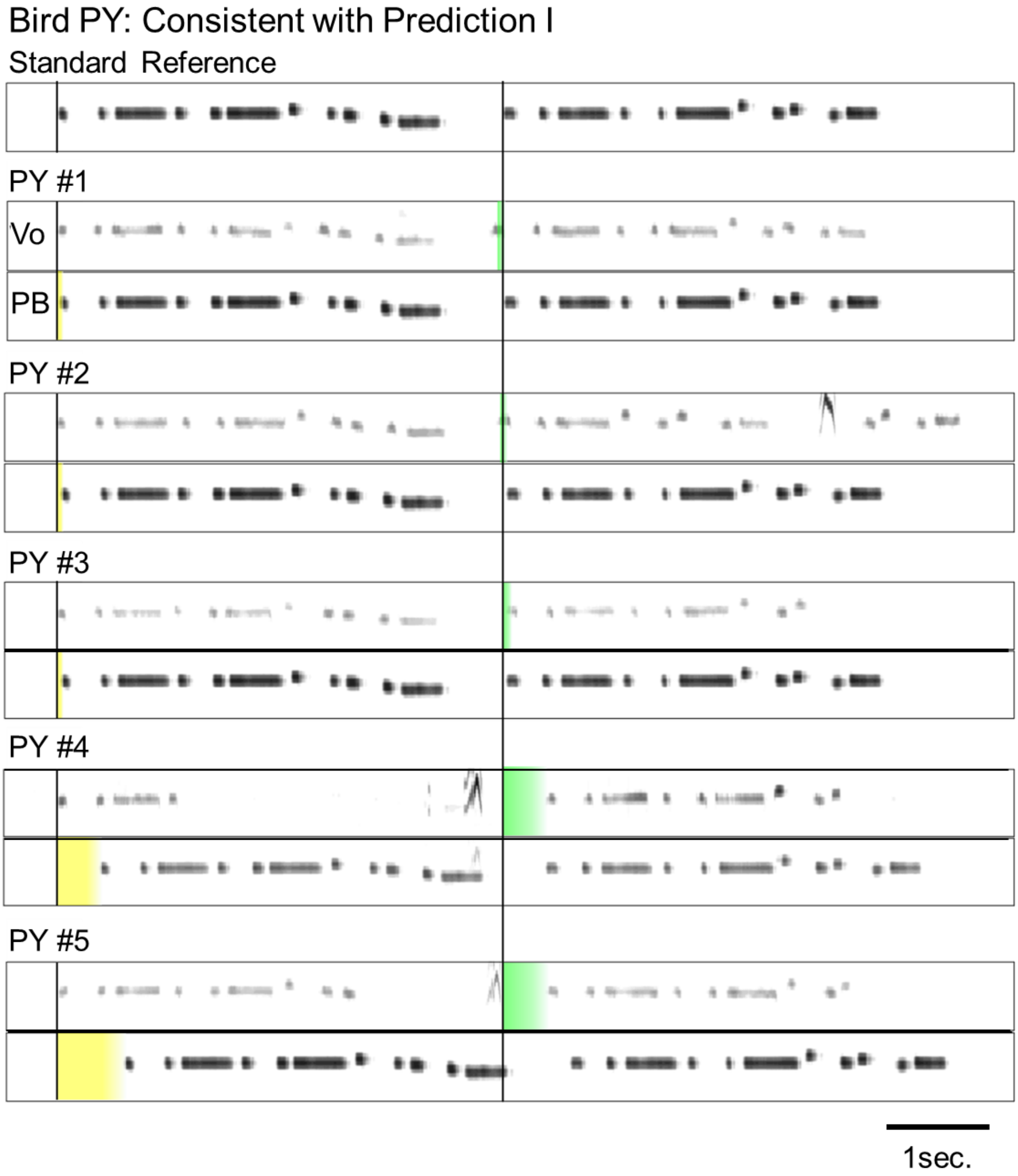

Similar recordings were obtained from bird PY.

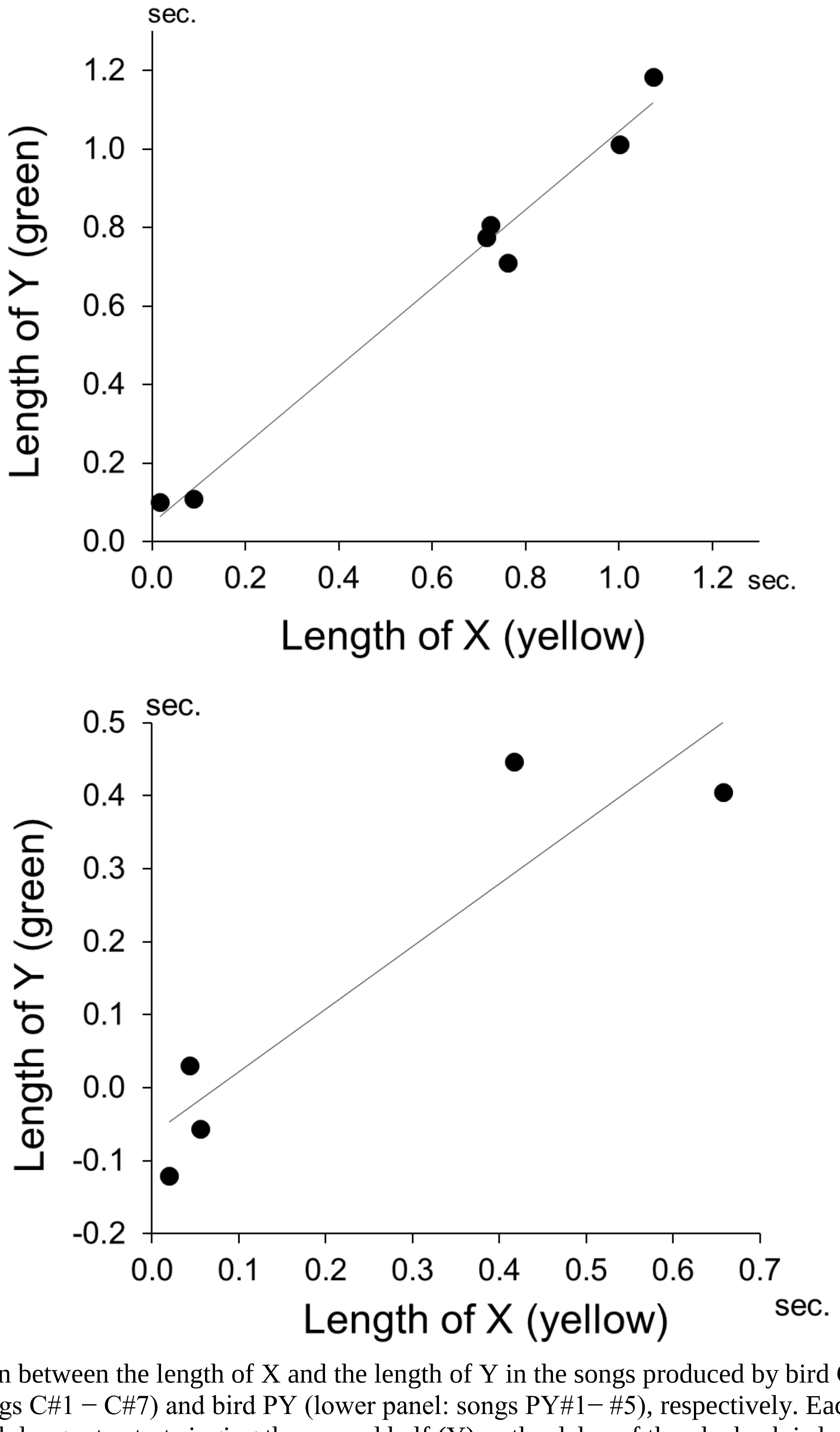

Correlation between the length of X and the length of Y in the songs produced by bird C (upper panel: songs C#1 − C#7) and bird PY (lower panel: songs PY#1− #5), respectively. Each bird waits much longer to start singing the second half (Y) as the delay of the playback is longer (X). Upper panel: $r = 0.991$ [95% CI = 0.939 − 0.999], $t = 16.68$, $df = 5$, $p < 0.001$; Lower panel: $r = 0.921$ [95% CI = 0.206 − 0.995], $t = 4.093$, $df = 3$, $p = 0.026$.

Bird PY: Consistent with Prediction II (a)

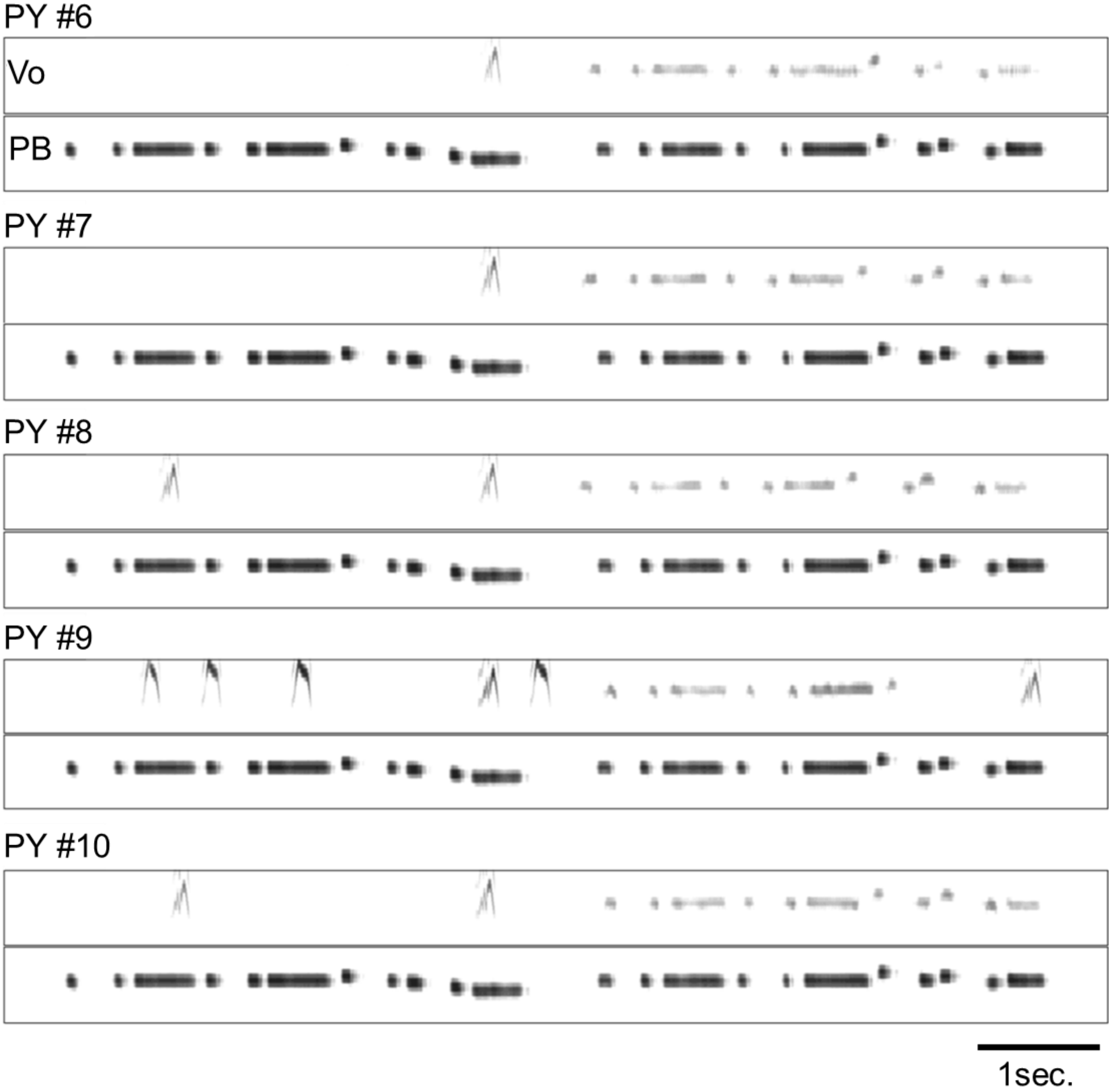

Bird PY sang songs following presentation of the playback sounds. He started singing and synchronized his vocal timing with the playback from the beginning of the second half, skipping the first half.

Note: the bird called at almost same time as when the 11th note was presented, as if he was adjusting his vocal timing in anticipation of singing the second half of the melody.

Bird PY: Consistent with Prediction II (b)

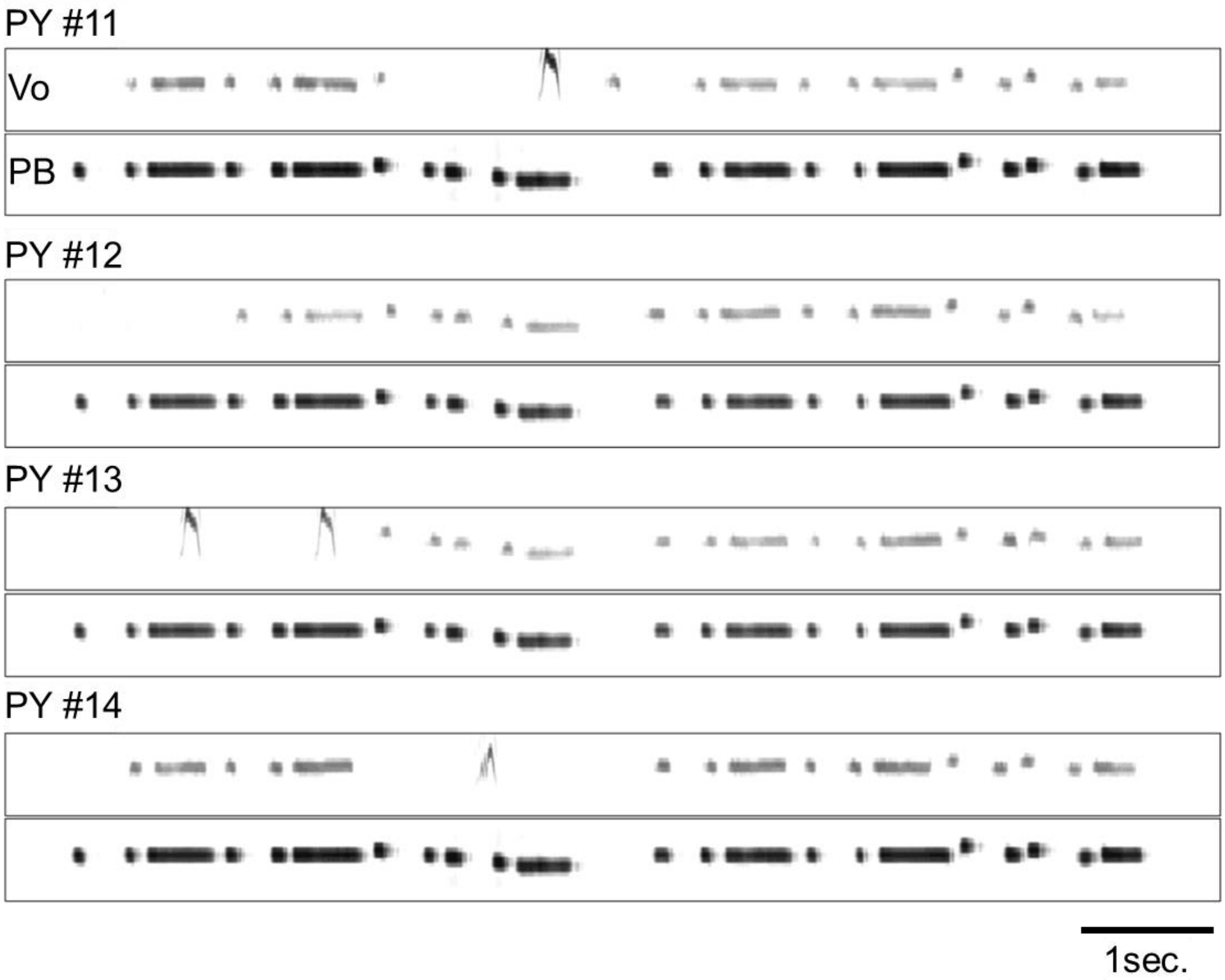

In these recordings, following the presentation of the playback sounds, bird PY started singing and synchronized his vocal timing with the playback from in the middle of the first half.

Bird PY: Consistent with Prediction III

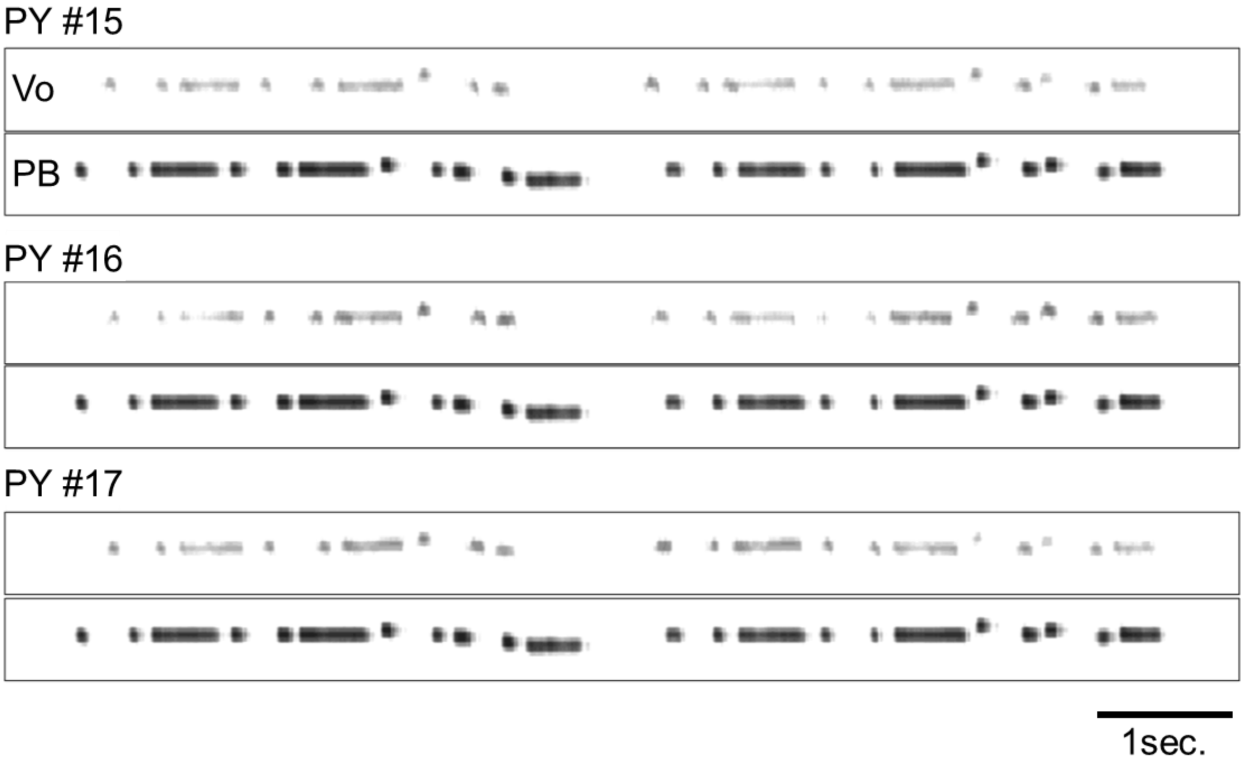

Bird PY sang songs following presentation of the playback sounds. In these recordings, he skipped the last two notes of the first half and synchronized his vocal timing with the playback at the beginning of the second half.

Bird PY: Other

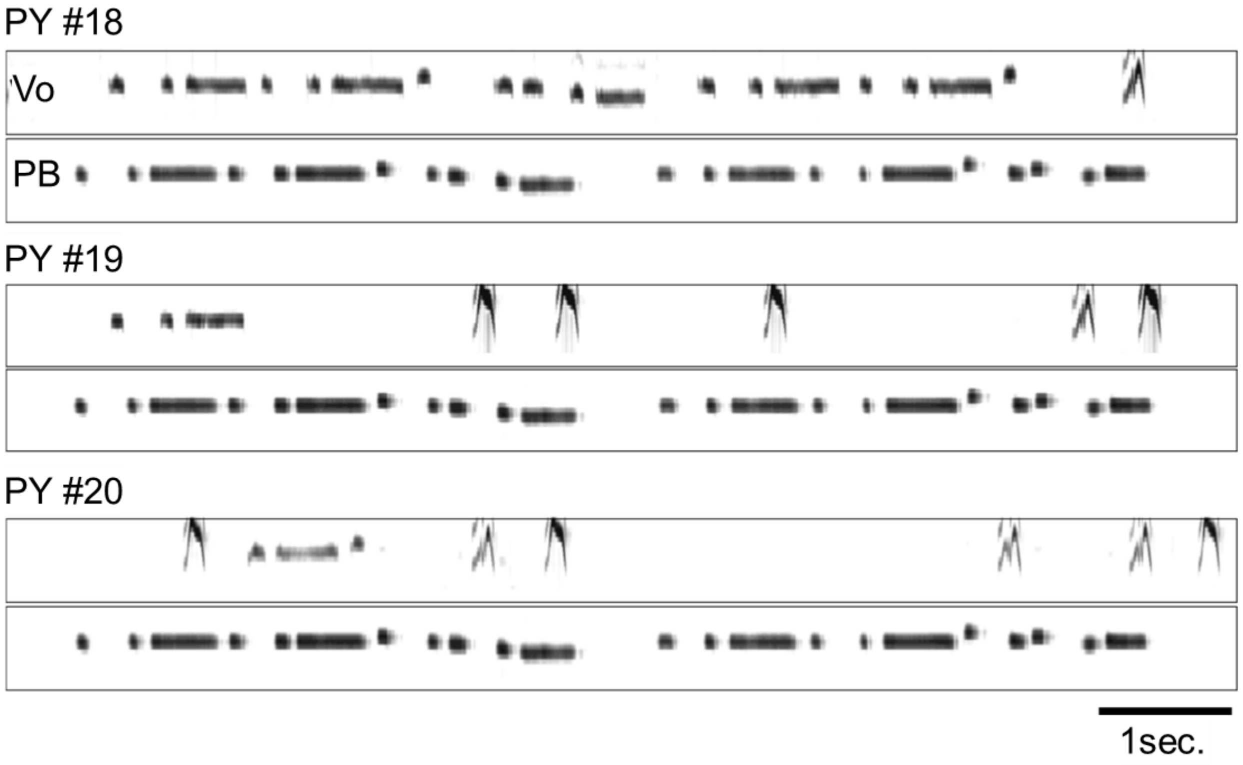

Bird PY sang songs following presentation of the playback sounds. The top panel shows that the bird sang the normal sequence of the melody with some irregular note intervals, as if he was ignoring the playback sound; then, he stopped singing in the middle of the second half (PY#18). Middle and bottom panels show that the bird sang a limited part of the first half and then stopped singing suddenly (PY#19 and PY#20). In these two cases, it is likely that, the bird attempted to adjust his vocal timing to the playback melody, but was not successful so he stopped singing.
